# *In silico* analysis of amino acid variation in human respiratory syncytial virus: insights into immunodiagnostics

**DOI:** 10.1590/0074-02760170013

**Published:** 2017-10

**Authors:** Claudemir Souza, Nilson IT Zanchin, Marco A Krieger, Adriana Ludwig

**Affiliations:** 1 Fundação Oswaldo Cruz-Fiocruz, Instituto Carlos Chagas, Laboratório de Genômica Funcional, Curitiba, PR, Brasil; 2 Instituto de Biologia Molecular do Paraná, Curitiba, PR, Brasil; 3 Universidade Federal do Paraná, Programa de Pós-Graduação em Biologia Celular e Molecular, Curitiba, PR, Brasil

**Keywords:** HRSV, genetic variability, diagnostics, antigen detection

## Abstract

**BACKGROUND:**

The highly contagious nature of human respiratory syncytial virus (HRSV) and the gravity of its infection in newborns and vulnerable adults pose a serious public health problem. Thus, a rapid and sensitive diagnostic test for viral detection that can be implemented upon the first appearance of symptoms is needed. The genetic variation of the virus must be considered for immunodiagnostic purposes.

**OBJECTIVES:**

To analyse HRSV genetic variation and discuss the possible consequences for capture immunoassay development.

**METHODS:**

We performed a wide analysis of N, F and G protein variation based on the HRSV sequences currently available in the GenBank database. We also evaluated their similarity with homologous proteins from other viruses.

**FINDINGS:**

The mean amino acid divergences for the N, F, and G proteins between HRSV-A and HRSV-B were determined to be approximately 4%, 10% and 47%, respectively. Due to their high conservation, assays based on the full-length N and F proteins may not distinguish HRSV from human metapneumovirus and other *Mononegavirales* viruses, and the full-length G protein would most likely produce false negative results due to its high divergence.

**MAIN CONCLUSIONS:**

We have identified specific regions in each of these three proteins that have higher potential to produce specific results, and their combined utilisation should be considered for immunoassay development.

Human respiratory syncytial virus (HRSV) is one of the main causes of lower respiratory infections, which result in severe burden, especially in children, the elderly and adults with chronic health problems. While there is not an updated epidemiological census, the incidence rate of HRSV infection was reported in a study by Nair et al. (2010). They estimated that 33.8 million children younger than five years old were infected with HRSV in 2005, and at least 3.4 million of these children were hospitalised due to severe complications.

HRSV is an enveloped virus belonging to the *Pneumoviridae* family and the *Mononegavirales* order (Afonso et al. 2016) that has a negative sense single-stranded RNA genome encoding 11 proteins (Hacking & Hull 2002). The nucleocapsid (N), fusion (F) and attachment (G) proteins have been the main targets of therapeutic and diagnostic investigations (Buraphacheep et al. 1997, Terrosi et al. 2007, Green et al. 2015). The N protein binds strongly to viral RNA to form a helical structure (Buraphacheep et al. 1997). The F protein mediates fusion of the viral membrane with the host cell membrane, delivers viral RNA to the cytoplasm, and fuses infected cell membranes with those of healthy cells, which results in syncytia formation (Hacking & Hull 2002). Together with the F protein, the HRSV G protein is responsible for binding host cell receptors and triggering infection (Melero et al. 1997). The G protein is a transmembrane glycoprotein that has an ectodomain containing two highly variable mucin-like regions (HVR1 and HVR2) that are also rich in glycosylation sites and immune epitopes (Melero et al. 1997).

Antigenic studies using monoclonal antibodies against the F and G proteins led to the initial distinction between the HRSV groups A and B. Subsequently, this virus was classified into genotypes based on the nucleotide variation of the G protein in the HVR2 region. Thus far, 14 genotypes have been identified for the HRSV-A group and 25 have been identified for the HRSV-B group (Hu et al. 2017, Zheng et al. 2017).

Early and accurate HRSV diagnosis is critical for preventive measures and patient treatment, and capture immunoassays can be performed faster than polymerase chain reaction (PCR)-based diagnostic assays. However, HRSV immunodiagnosis remains inefficient and problematic (Brendish et al. 2015, Koo et al. 2017) mostly because of false positive results from cross-reactivity with similar proteins of other viruses and false negative results due mostly to high viral population variation. Taking this into account, the search for specific diagnostic targets must consider the genetic variation of the target protein and the similarity to proteins of related viruses. For this purpose, we have analysed the HRSV N, F and G proteins currently available in the GenBank database. Specifically, we have examined their amino acid (aa) variation within the HRSV taxId and their similarity to other respiratory viruses and have evaluated their potential use in or exclusion from immunodiagnostic applications.

## MATERIALS AND METHODS

*Sequence data, phylogenetic and divergence analyses* - The aa sequences of the HRSV N, F and G proteins were retrieved from the GenBank database. Since there is a substantial number of partial sequences, we used all sequences that included complete aa coverage and partial entries containing a minimum of 70% sequence coverage (through November 2016). We initially retrieved 1102 sequences for the N protein, 1426 for the F protein and 701 for the G protein. The sequence datasets were aligned using the MUSCLE algorithm of MEGA 7.0 software (Kumar et al. 2016).

The high number of retrieved sequences made it impossible to generate a visually informative phylogeny tree. In addition, due the excessive redundancy of the datasets, we selected a defined number of representative sequences to evaluate the variability of the HRSV proteins at the aa level. Initial phylogenetic trees for the N and F proteins that included all sequences retrieved from GenBank were generated using the neighbour joining method with a 500 replicate bootstrap test. Representative sequences of the major clades were randomly chosen to construct the final phylogenies and for further analyses. Sequences that presented high divergence, which were evident by long branches, were also chosen. Moreover, strain reference sequences available in the NCBI taxonomy database (http://www.ncbi.nlm.nih.gov/taxonomy) were also included. Finally, sequences from other related viruses were used as the outgroup to root the trees. For the G protein, high diversity is represented by the various genotypes that have been described. Thus, we opted to use one representative sequence from each genotype (with a minimum of 70% aa sequence coverage). Due to the high divergence of the G protein and consequential low similarity and poor alignment with its homologues from other viral species, we opted to present an unrooted tree. The G protein alignment was obtained by PSI-Coffee, which was designed to align distantly related proteins (Kemena & Notredame 2009).

The final phylogeny for each protein was constructed by the maximum likelihood (ML) method using the aa substitution model indicated by the model selection. Bootstrap tests with 1,000 replicates for the N and F proteins and 500 replicates for the G protein were used to assess the reliability of the branches. Amino acid sequence divergences were obtained using p-distance, which calculates the number of aa differences per site between sequences, using the pairwise deletion option. All divergences and phylogenetic analyses were assessed using MEGA 7.0 software (Kumar et al. 2016).

*HRSV similarity to other viruses* - To evaluate the possible cross-reactivity of the HRSV in immunological tests, we investigated the similarity of the HRSV N, F and G proteins with their homologous proteins from other viruses that infect humans using NCBI BLASTP searches. Since there are hundreds to thousands of entries for each virus, we used a strategy based on several rounds of taxon exclusion to access more distantly related viruses. We performed continuous taxon exclusion with a low stringency e-value cut-off of 1 until no additional significant hits were found. Additionally, BLASTP searches (e-value cut-off of 100) specifically against *Mononegavirales* respiratory viruses were performed to confirm the absence of similarity. The query sequences for the N and F proteins were P03418.1 and P03420.1, respectively. For the G protein, P03423.1 and O36633.1, corresponding to sequences from HRSV-A and HRSV-B, respectively, were used due to the high divergence found between these groups. The protein sequences corresponding to the best hits from each virus were aligned to the HRSV-A and HRSV-B sequences using PSI-Coffee (Kemena & Notredame 2009).

## RESULTS AND DISCUSSION

*The N protein* - N proteins are among the most abundant viral proteins of some *Mononegavirales* viruses, and they usually elicit a strong, and long-lasting humoral immune response in patients. N proteins are of special interest because they could be used for the development of simple and rapid laboratory diagnostic assays for direct virus detection in clinical specimens (Petraityté-Burneikiené et al. 2011). These proteins also have strong species-specific patterns and can be good markers for early diagnosis (Li et al. 2015). The HRSV N protein is a 391-aa protein that homomultimerizes into a decameric ring that functions as a scaffold around which viral RNA associates to form nucleocapsids (Tawar et al. 2009, El Omari et al. 2011).

**Fig. 1:**
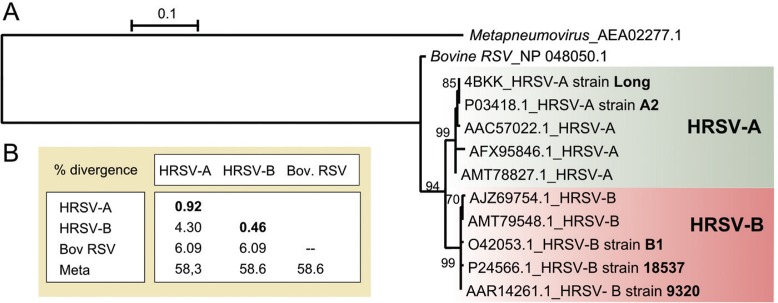
: analysis of the human respiratory syncytial virus (HRSV) N protein. (A) Maximum likelihood tree based on the JTT + I model. The bootstrap values are shown next to the branches (only greater than 50). The tree is drawn to scale, and the branch lengths are measured as the number of substitutions per site. The analysis involved 10 HRSV amino acid (aa) sequences, which are identified by their GenBank accession number and NCBI subgroup (in bold). The N protein of the bovine respiratory syncytial virus and human metapneumovirus were used as outgroup*.* (B) Mean aa divergence (based on p-distance) found between the major clades HRSV-A, HRSV-B and bovine respiratory syncytial virus (Bov RSV) and human metapneumovirus (Meta). The mean amino acid divergence found within the groups is shown in bold.

The HRSV N protein is highly conserved. It displays only a 1.89% overall mean aa divergence among the 1102 aa sequences analysed in this work. The maximum pairwise divergence value is 5.38% between the AJZ69754 (isolate = “VN-817-8/10”, Vietnam) and AFX95846.1 (strain = “RSVA/GN435/11”, South Korea) sequences. Alignment of the N protein sequences shows high conservation across the entire protein (Supplementary data, Fig. 1). The N protein phylogeny (Fig. 1A), which shows two major well-supported clades corresponding to HRSV-A and HRSV-B, revealed that the N protein is highly conserved as indicated by the short branch lengths. The mean divergence within the A and B groups is less than 1%, and the mean divergence between the groups is 4.3% (Fig. 1B).

To investigate the similarity of the HRSV N protein with its homologous counterparts from other human-infecting viruses, we performed serial NCBI BLASTP searches, which are summarised in Table I. Although this protein is highly conserved within HRSV, it shows significant similarity (61%) with only the N protein of the human metapneumovirus (HMPV), another *Pneumoviridae* respiratory virus. Based on sequence comparison, no significant hits were recovered for the HRSV N protein with other human-infecting *Mononegavirales* viruses*,* although it has been predicted to show secondary structure conservation (Barr et al. 1991).

**TABLE I:** Summary of BLASTP results for N, F and G proteins against the NCBI non-redundant protein database. Only the best hit against human-infecting viruses were considered

Order	Family	Genus	Species	Extension	e-value	Identity	Similarity	Ac Number	Alignment position
N protein									
Mononegavirales	*Pneumoviridae*	*Metapneumovirus*	*Human metapneumovirus*	99%	4e-101	42%	61%	AEA02277.1	1-388
F protein									
Mononegavirales	*Pneumoviridae*	*Metapneumovirus*	*Human metapneumovirus*	88%	2e-119	36%	59%	AGL74059.1	14-519
	*Paramyxoviridae*	*Respirovirus*	*Human parainfluenza virus 3*	66%	8e-09	19%	40%	NP_067151.1	133-516
		*Henipavirus*	*Nipah virus*	19%	0.055	29%	51%	AAY43915.1	139-251
		*Henipavirus*	*Hendra virus*	75%	0.14	21%	38%	AEQ38114.1	139-569
		*Morbillivirus*	*Measles virus*	17%	0.061	25%	54%	ABY58017.1	115-214
G protein									
No significant similarity to other Human-infecting viruses									

Extension: the percentage of alignment length with the query sequence; Ac: Genbank accession number; alignment position: the amino acid position of the alignment extension in relation to the query.

The similarity between the N proteins of HRSV and HMPV mainly lies in the first 31 aa_s_ and in the region from aa 160 to the C-terminal end (Supplementary data, Fig. 2), which could implicate in possible serological cross-reactivity. Consequently, polyclonal antibodies against this protein or monoclonal antibodies against these regions are not appropriate for HRSV diagnosis. In fact, cross-reactivity between the N proteins of these viral species has already been described for polyclonal antibodies and for monoclonal antibodies against these two mentioned regions (Zhang et al. 2015). Alternatively, monoclonal antibodies against the region between aa_s_ 30-160 could specifically identify HRSV since this region is conserved only among HRSV sequences.

*The F protein* - The HRSV fusion glycoprotein F is a type I integral membrane protein that is synthesised as a 574-aa precursor, which is processed by a furin-like protease to excise a 27-residue glycosylated peptide (pep27), giving rise to the F2 (N-terminal region) and F1 (C-terminal region) polypeptides. These subunits are connected by two disulfide bonds and form a protomer that oligomerizes to form the mature trimeric F protein (Hacking & Hull 2002, McLellan et al. 2013). The presence of these two furin sites separated by pep27 is a specific feature of the F protein from HRSV (Bolt et al. 2000), although the basic F protein characteristics are shared with other *Pneumoviridae* and *Paramyxoviridae* family members. During cell entry, F glycoproteins undergo a conformational change that brings the viral and cellular membranes into proximity, ultimately leading to their fusion (Swanson et al. 2011).

A total of 1426 aa F protein sequences were ana- lysed. The overall mean aa divergence is 4.66%, and the maximum pairwise divergence value is 15.98% between the AHA61614.1 (isolate = “B6-9918”, Taiwan) and AHA61607.1 (isolate = “A10-6030”, Taiwan) sequences. Alignment of the selected F protein sequences is shown in Supplementary data, Fig. 3. The signal peptide presents the highest divergence (mean of 35%), while the F1 and F2 subunits appear to be more conserved (divergence mean of 3.3% and 4.6%, respectively).

**Fig. 2:**
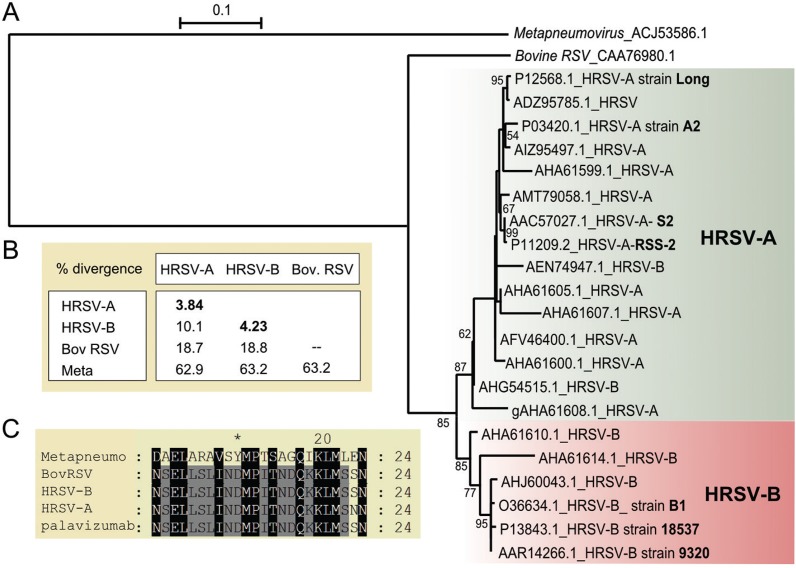
: analysis of the human respiratory syncytial virus (HRSV) F protein. (A) Maximum likelihood tree based on the JTT + G model. The bootstrap value is shown next to the branch (only greater than 50). The tree is drawn to scale, and the branch lengths are measured as the number of substitutions per site. This analysis involved 22 HRSV amino acid (aa) sequences, which are identified by their respective GenBank accession number and NCBI subgroup (in bold). The F protein from bovine respiratory syncytial virus and human metapneumovirus were used as outgroups. (B) Mean aa divergence (based on p-distance) found between the major clades HRSV-A and HRSV-B and the outgroup sequences from bovine respiratory syncytial virus (Bov RSV) and human metapneumovirus (Meta). The mean aa divergence found within the groups is shown in bold. (C) Amino acid sequence alignment of the palivizumab-specific antibody epitope (254-277 aa). One sequence from HRSV-A, HRSV-B, bovine respiratory syncytial virus and human metapneumovirus is represented in the alignment.

The F protein phylogeny, shown in Fig. 2A, was constructed based on 22 selected HRSV sequences. As expected, two well-supported groups are evident from the F protein tree, reflecting the separation of HRSV-A and HRSV-B. The mean divergence found within each group is nearly 4%, while the mean divergence between groups A and B is 10.1% (Fig. 1B). Phylogeny analysis revealed that two sequences that were deposited in GenBank as belonging to the HRSV-B group (AEN74947.1 and AHG54515.1) felt in the HRSV-A clade. Since HRSV classification is based mostly on the G protein, a possible explanation for this finding is the occurrence of recombination between co-circulating HRSV-A and HRSV-B. However, although recombination is common for some types of viruses, it is rare for HRSV, and it is most likely that these potential recombinants are due to either PCR or sequence assembly artifacts (Tan et al. 2012).

Although the HRSV F protein is less conserved than the HRSV N protein, it presents significant similarity with F proteins of a higher number of viruses (Table I). The F protein most similar to that of HRSV is from HMPV, sharing 36% identity and 59% similarity and length coverage of 88% of the aa sequence. Two segments of the F protein are more conserved in these viruses (Supplementary data, Fig. 4), including a region of 29 aa_s_ (positions 36 to 64 of the HRSV F protein) that has 53% identity and 84% similarity and a central region of 16 aa_s_ that has 81% identity and 100% similarity (positions 303 to 318 of the HRSV F protein). The most divergent regions are the signal peptide and the region from aa_s_ 100 to 130.

According to the F protein BLASTP scores, the viruses identified as having similar F proteins are human parainfluenza virus 3*,* the Nipah virus*,* the Hendra virus and the measles virus. All of these viruses are from *Paramixoviridae*, a related family of the *Mononegavirales* order (Afonso et al. 2016). However, the similarity and/or extensions of the BLAST alignment results are not high. The alignments are shown in Supplementary data, Figs 5-8 and illustrate only a few short regions that are well-conserved. No additional human-infecting viruses show significant similarity with the HRSV F protein. Nevertheless, polyclonal antibodies against the HRSV F protein could produce cross-reactivity to these viruses and should be avoided for diagnostic purposes. We identified only two short regions of the HRSV F protein (aa_s_ 97-109 of F2 and 417-428 of F1) that are highly divergent in relation to other viruses and could thus be used for specific monoclonal antibody development to avoid cross-reactivity.

We also closely analysed the F1 region between aa_s_ 254-277, which corresponds to the epitope of the palivizumab antibody (MEDI-493, Synagis, MedImmune Inc., Gaithersburg, MD). Palivizumab has been used since 1998 for preventing infection in children who have a substantial risk of developing severe forms of HRSV disease (Eiland 2009). The palivizumab epitope consists of a structural helix-loop-helix motif that has discontinuous residues within a 20-residue linear peptide (Corti et al. 2013, McLellan 2015). Among the *Mononegavirales* human-infecting species, only the HMPV F protein is relevantly conserved in this region with the HRSV F protein (Fig. 1C), as the linear sequence alignment shows 37% identity and 58% similarity. However, the possible antibody contacts with this type of discontinuous epitope make it difficult for reliable cross-reaction prediction.

*The G protein* - The HRSV G protein has a variable sequence length ranging from 282 to 321 aa_s_ depending on the genotype and has no sequence similarity with other *Mononegavirales* attachment proteins (Cui et al. 2013). It is highly glycosylated, containing 30-40 O-linked glycans and 4-5 N-linked glycans, which potentially constitutes up to 60% of the G protein molecular mass (Cui et al. 2013, McLellan et al. 2013).

The overall mean aa divergence among the 701 G protein sequences analysed is 27.6%, which is consistent with the expected low conservation. The maximum pairwise divergence value is 61.4% between the AIF71060.1 (HRSV-B, isolate = “MI_B_55_12_12”, Italy) and P27021.1 (HRSV-A, strain RSB642) sequences. As mentioned above, the high divergence found for the G protein is represented by numerous distinct genotypes, with 14 genotypes assigned to HRSV-A (NA1-NA4, GA1-GA7, SAA1, CB-A, and ON1) and 25 genotypes for assigned to HRSV-B (BA1-BA12, BA-C, SAB1-SAB4, GB1-GB4, URU1-2, CB-B, CB1) (Hu et al. 2017, Zheng et al. 2017). The genotypes that had only short, partial sequences available (HRSV-A: GA6 and SAA1; HRSV-B: BA6, BA8, BA11, SAB2 and URU1) were excluded from our analyses, and the phylogeny was inferred using 32 G protein sequences representing different genotypes (12 genotypes from HRSV-A and 20 from HRSV-B).

**Fig. 3:**
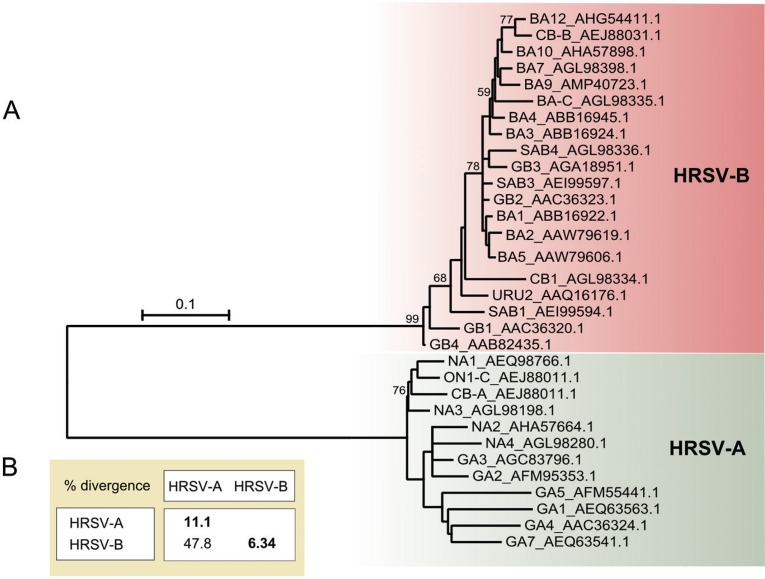
: analysis of the human respiratory syncytial virus (HRSV) G protein. (A) Maximum likelihood unrooted tree based on the JTT + G model. The bootstrap value is shown next to the branch (only greater than 50). The tree is drawn to scale, and the branch lengths are measured as the number of substitutions per site. The analysis involved 32 HRSV amino acid (aa) sequences, which are identified by their genotype followed by their GenBank accession number. (B) Mean aa divergence (based on p-distance) found between the major clades HRSV-A and HRSV-B. The mean aa divergence found within the groups is shown in bold.

Fig. 3A shows the unrooted phylogeny of the HRSV G protein, which was constructed based on the entire protein. Presumably, two clades separate the HRSV-A and HRSV-B sequences, and the long branches suggest high divergence among the sequences. The relationships among the genotypes are not clear since the bootstrap values for most nodes are very low (< 50). This lack of resolution is not relevant to the main purpose on this work, and it may reflect the non-dichotomous nature of the HRSV lineage split. The mean divergence found within the A and B groups is 11.1% and 6.34%, respectively, and the mean divergence between these groups is 47.8% (Fig. 3B), which are similar to the values described in previous studies (Melero et al. 1997).

**Fig. 4:**
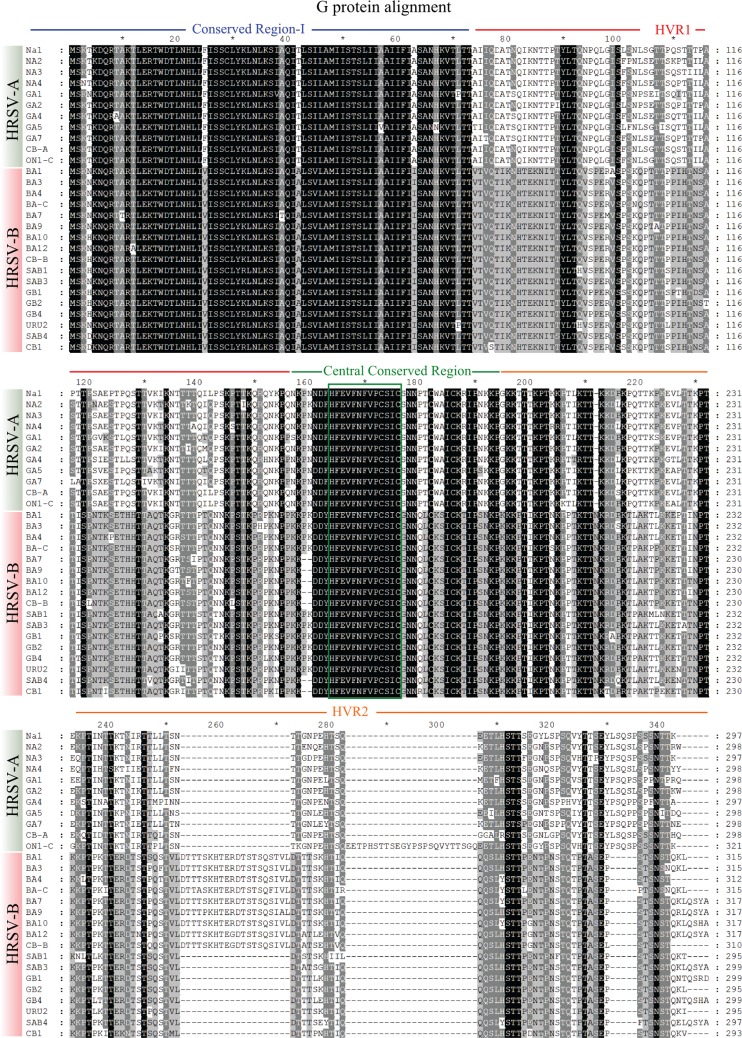
: amino acid (aa) alignment view of the G protein from the selected human respiratory syncytial virus (HRSV) sequences representing different genotypes. The sequences are identified by their genotype. The GenBank accession number of the sequences can be found on the G protein phylogeny. Of the 32 selected sequences, incomplete sequences were excluded (GA3, GB3, BA2 and BA5). The HRSV-A and HRSV-B sequences are specified on the left. The conserved region I, HVR1, the central conserved region and HVR2 are indicated by blue, red, green and orange lines, respectively. The green box denotes the 13-aa segment (164-176 aa) from the central conserved region that is identical in all HRSV genotypes.

Fig. 4 shows the alignment of the selected G protein sequences. The G protein can be divided into four regions that represent various levels of divergence: conserved region I, HVR1, a central conserved region and HVR2. A summary of aa divergence from these regions is presented in Table II. The low variation of conserved region I and the central conserved region contrasts with the high variation of the remaining G protein regions. The central conserved region has 13 aa_s_ that are identical in all the sequences analysed. Between groups A and B, the mean divergence for HVR1 and HVR2 is 67.1% and 57.3%, respectively. Variation in these regions within group A is 14.4% and 18.4%, respectively. The sequence divergence is lower in group B, with a mean of 7.61% for HVR1 and 11% for HVR2. Differences in HVR1 are caused mainly by aa substitutions, while the differences in HVR2 are caused by both aa substitutions and the insertion and deletion of sequence segments (Cui et al. 2013). Thus, these regions are not suitable targets for prevention (Tan et al. 2013) and diagnosis since they could generate false negative results. In contrast, the 13-aa central conserved region would be an excellent target for diagnosis and prevention. This region (residues 164-176) is unglycosylated and universally conserved among all clinical isolates (Maifeld et al. 2016). In fact, monoclonal antibodies against this region have been shown to successfully block HRSV infection and disease (Jorquera et al. 2016).

**TABLE II:** Amino acid divergence pattern of the G protein distinct regions among human respiratory syncytial virus (HRSV) sequences

Region	Conserved region I	HVR1	Central conserved region	HVR2
Position	1-73 aa	74-156 aa	157-194 aa	195- last aa
Overall mean divergence	7.7%	38%	15.5%	35%
Higher pairwise distance	16.44%	71.1%	31.6%	66.3%
Mean divergence within HRSV-A group	2.19%	14.4%	1.91%	18.4%
Mean divergence within HRSV-B group	2.10%	7.61%	0.33%	11.0%
Mean divergence between HRSV-A and HRSV-B groups	13.47%	67.1%	30.5%	57.3%

The analysis involved 28 amino acid (aa) sequences (11 from HRSV-A and 17 from HRSV-B). HVR: highly variable mucin-like regions.

The high divergence of the HRSV G protein is also reflected in its divergence from other viruses. No significant similarities were found to other human-infecting viruses (Table I). The absence of linear and structural similarity (Melero et al. 1997) of the HRSV G protein to other *Mononegavirales* attachment proteins probably prevents cross-reactivity.

*Genetic variability and HRSV diagnostic tests* - Available HRSV conventional diagnostic methods include viral culture, viral antigens detection by direct or indirect immunofluorescent (IF) or enzyme-linked immunosorbent assay (ELISA) and viral nucleic acid detection by reverse transcription-PCR (RT-PCR) (Popow-Kraupp & Aberle 2011). Although these methods are useful, they require costly equipment and reagents and/or trained operators and can be time consuming, restricting their use to large centres since they are not available in all hospitals and cities. Additionally, clinical sample quality has a direct impact on the sensitivity and specificity of these assays (Popow-Kraupp & Aberle 2011).

An ideal test for this disease should be rapid, inexpensive, easy to handle and compatible with use in remote areas that have no laboratory infrastructure. This can be achieved using immunochromatographic membrane assays designed with capture antibodies optimised for HRSV proteins, which could be the first test performed in acutely-infected patients, allowing for quick contention measures to be taken. A second confirmatory test could be subsequently performed, preferable for genotype identification and for treatment decisions.

The challenges of HRSV immunodiagnostics are related to the false positive results caused by cross-reactivity and false negative results due to viral population variation or low-quality clinical samples. We analysed the HRSV proteins reported to be the most immunogenic in terms of their genetic variation among the HRSV genotypes and their similarity to other respiratory viruses and evaluated their potential use in or exclusion from diagnostic applications. It is important to note that the analyses described herein were performed using linear alignment of the aa sequences, which was meant to be a starting point for the development of diagnostic monoclonal antibodies and to be complemented by other methods, including further epitope mapping and three-dimensional structural protein analysis.

HRSV proteins have various levels of genetic variation, with the N protein being extremely conserved and the F protein showing intermediate variation. The G protein, however, plays central role in the dynamics of HRSV genetic variation. This protein has a high capacity for harbouring long insertions without affecting its function, a feature that is related to the structural flexibility of its mucin-like domains, which allows for fast evolutionary changes. Changes in these domains may alter the glycosylation pattern and thus change the viral antigenic properties (Cui et al. 2013). Due to its high genetic and antigenic divergence, determined from numerous sequences isolated worldwide, the G protein has become a model system for studies on epidemiology and pathogen evolution (Cane 2007). The appearance of the novel genotypes may help HRSV escape immunity acquired from previous infections, influence infection severity and cause novel outbreaks (Cui et al. 2013).

Based on our analyses, we suggest that the conserved central region of the G protein and the specific regions of the F and N proteins described above seem the most suitable segments for the development of monoclonal antibodies for diagnostic applications. These antibodies could be used for capture of viral particles and viral proteins that are recommended for the acute phase of infection. Cross-reactivity could most likely occur with the N and F proteins of HMPV, a common, related respiratory pathogen found worldwide. This false positive result could be easily ruled out by a parallel G protein negative result, which could be interpreted as an infection by other *Mononegavirales* viruses. Thus, combined use of these regions could provide the basis for improving HRSV immunodiagnosis by diminishing both false negative results and cross-reactivity.
