# Supplementary material for: In silico analysis of amino acid variation in human respiratory syncytial virus: insights into immunodiagnostics
Source: Mem Inst Oswaldo Cruz. 2017 Oct;112(10):655–63. doi: 10.1590/0074-02760170013 (PMC5607514; doi:10.1590/0074-02760170013)
Supplement: Supplementary file 1 [file 0074-0276-mioc-112-10-0655-suppl01.pdf]

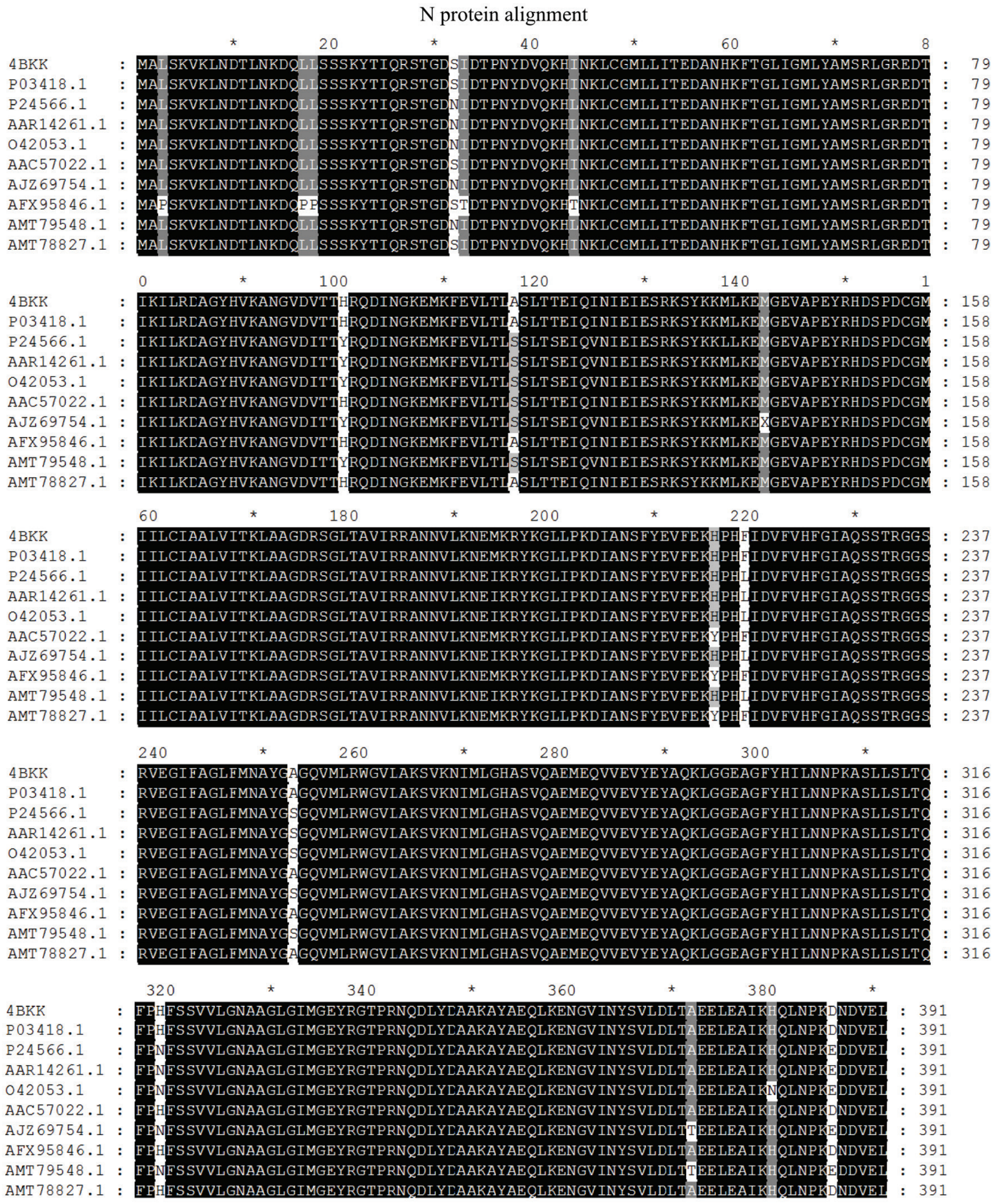


Fig. 1: amino acid (aa) alignment view of the N protein from the selected human respiratory syncytial virus (HRSV) sequences showing high conservation throughout the entire protein. The sequences are identified by their GenBank accession numbers.

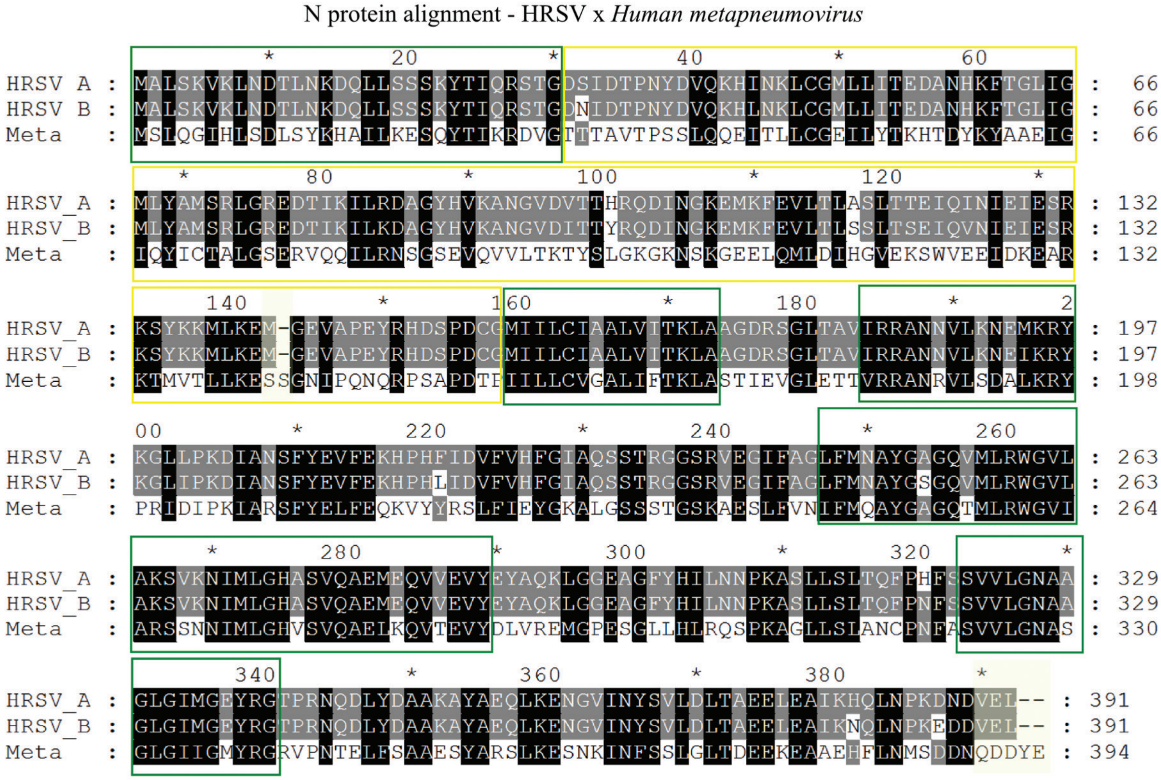


Fig. 2: amino acid (aa) alignment view of the N protein from the selected human respiratory syncytial virus-A (HRSV-A) (4BKK), HRSV-B (O42053.1) and human metapneumovirus (AEA02277.1) sequences. The green boxes show conserved regions. The yellow box shows the most divergent region. The ivory, filled boxes represent the unreliable alignment regions according to the PSI-Coffee result and should not be considered.

F protein alignment

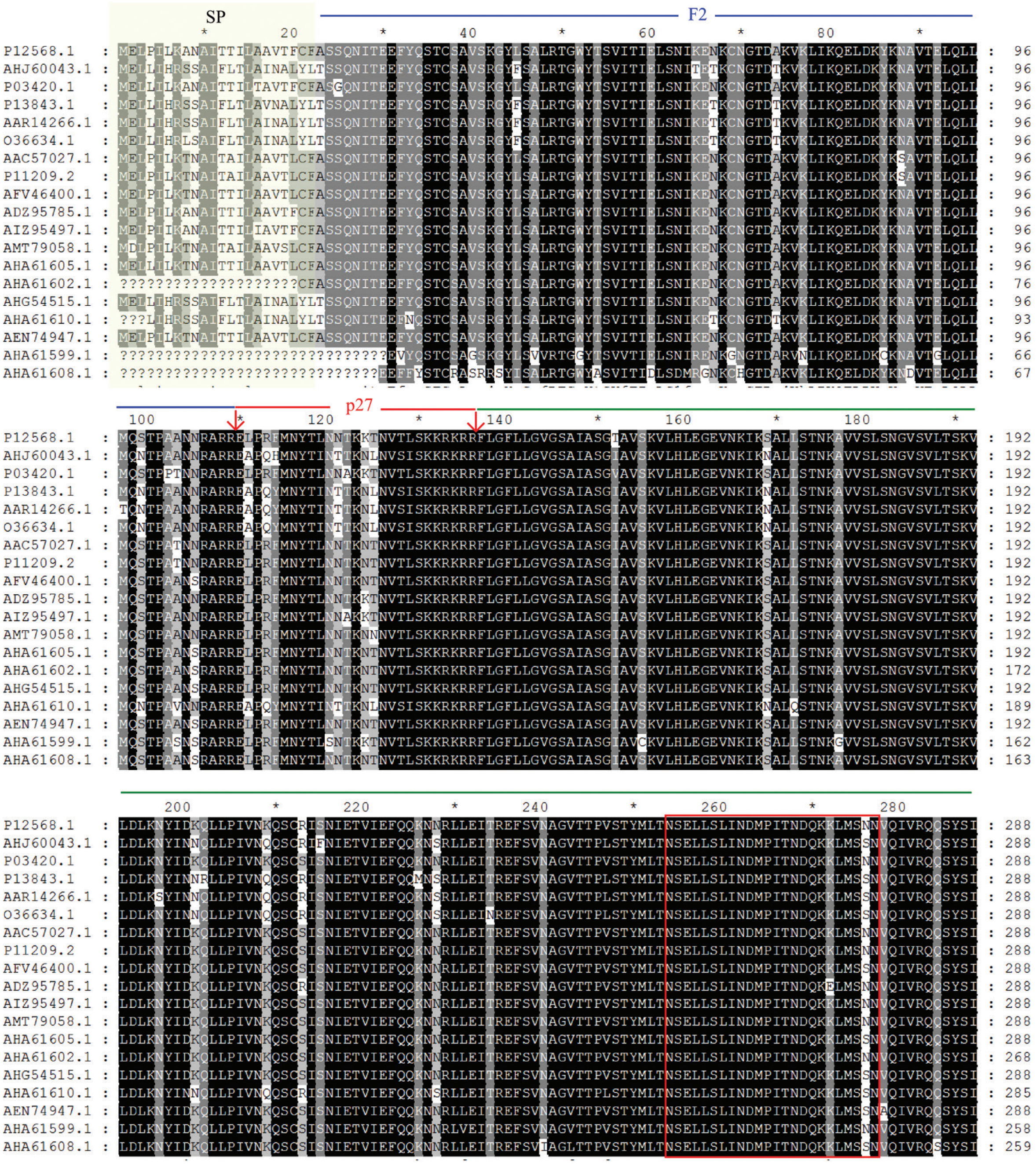

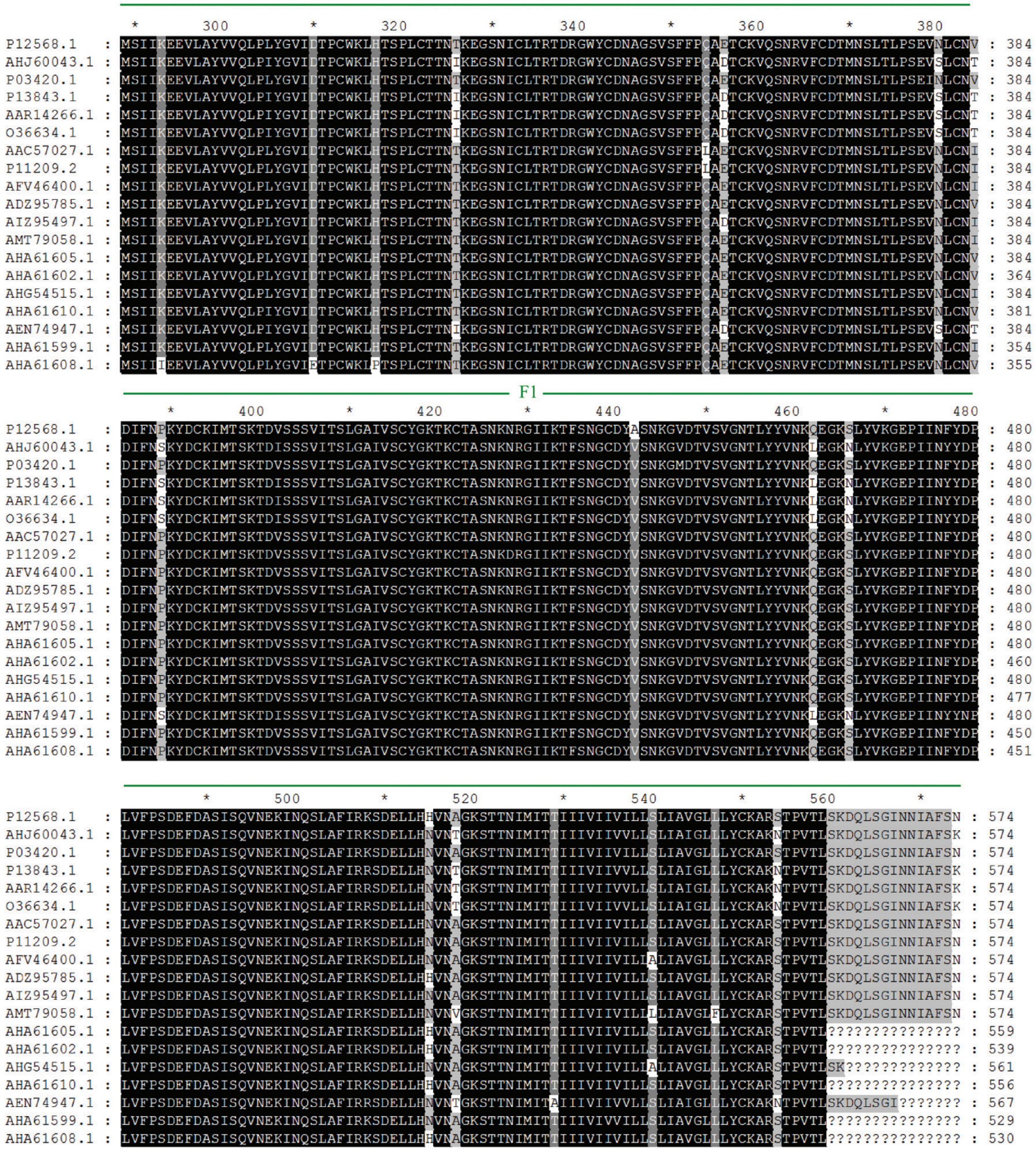

Fig. 3: amino acid (aa) alignment view of the F protein from the selected human respiratory syncytial virus (HRSV) sequences showing conservation throughout the entire protein. The sequences are identified by their GenBank accession numbers. Missing alignment data is represented as a question mark (?). The ivory, filled box on the N-terminal region represents the signal peptide (SP). The F2, p27 and F1 regions are indicated by blue, red and green lines, respectively. The palivizumab-specific antibody epitope sequence is represented by a red box.

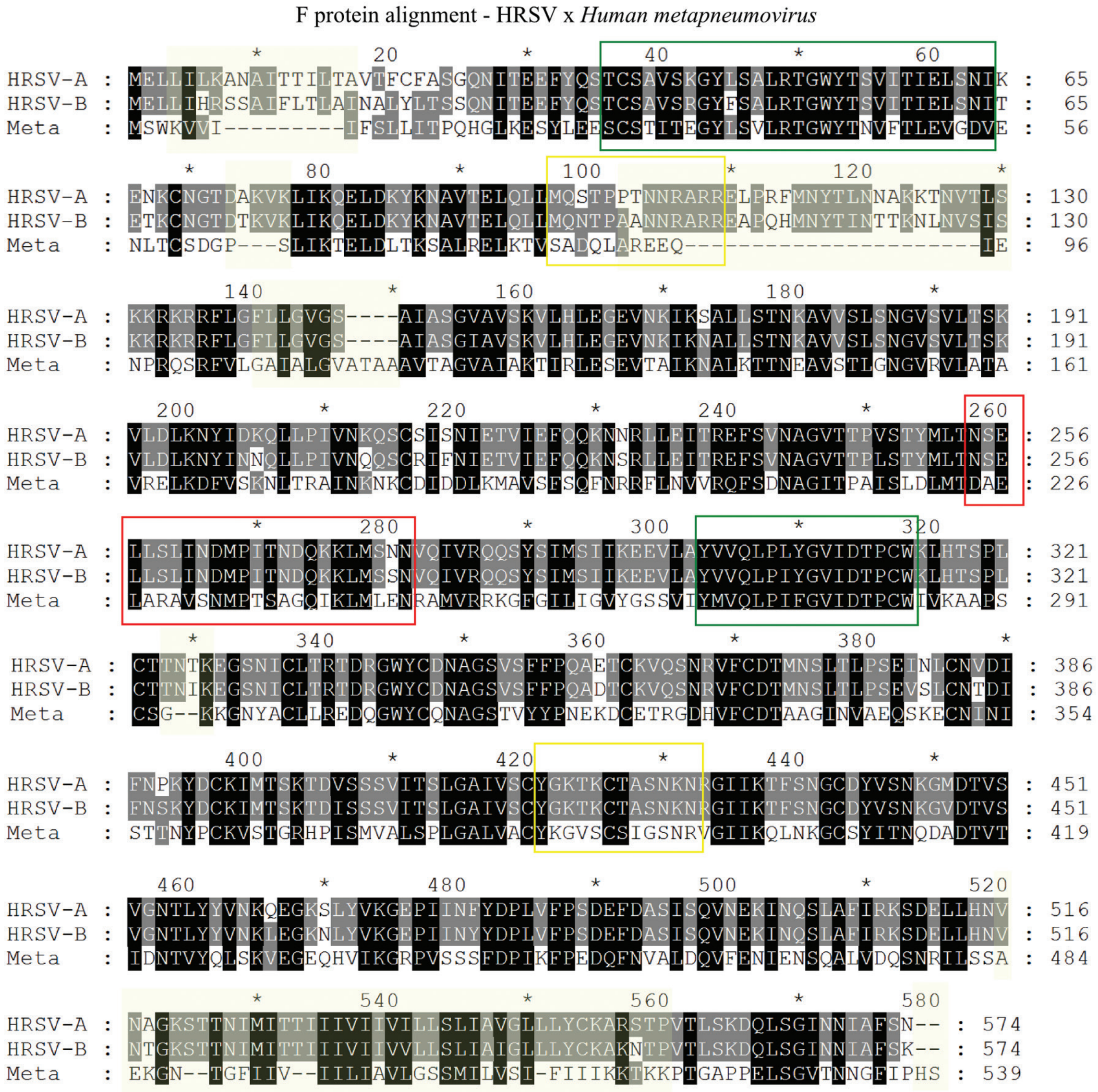


Fig. 4: amino acid (aa) alignment view of the F protein from the selected human respiratory syncytial virus-A (HRSV-A) (P03420.1), HRSV-B (AHJ60043.1) and human metapneumovirus (AAQ67695.1) sequences. The green boxes show conserved regions, and the yellow boxes show the possible targets (positions 97-109 and 417-428). The palivizumab-specific antibody epitope sequence is represented by a red box. The ivory, filled boxes represent the unreliable alignment regions according to the PSI-Coffee result and should not be considered.

F protein alignment - *Human parainfluenza virus 3*

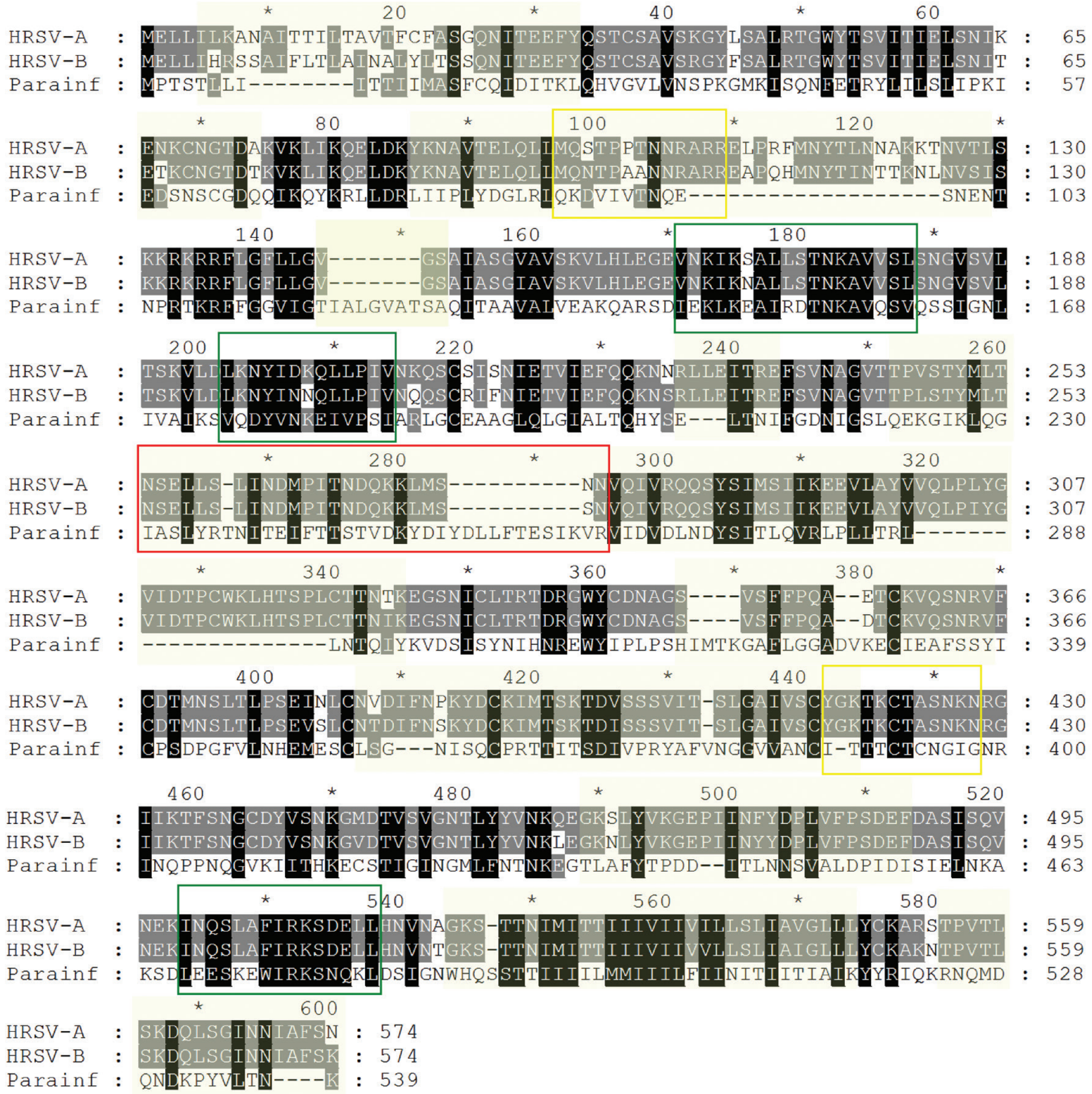


Fig. 5: amino acid (aa) alignment view of the F protein from the selected human respiratory syncytial virus-A (HRSV-A) (P03420.1), HRSV-B (AHJ60043.1) and human parainfluenza virus 3 (NP_067151.1) sequences. The green boxes show conserved regions, and the yellow boxes show the possible targets (positions 97-109 and 417-428). The palivizumab-specific antibody epitope sequence is represented by a red box. The ivory, filled boxes represent the unreliable alignment regions according to the PSI-Coffee result and should not be considered.

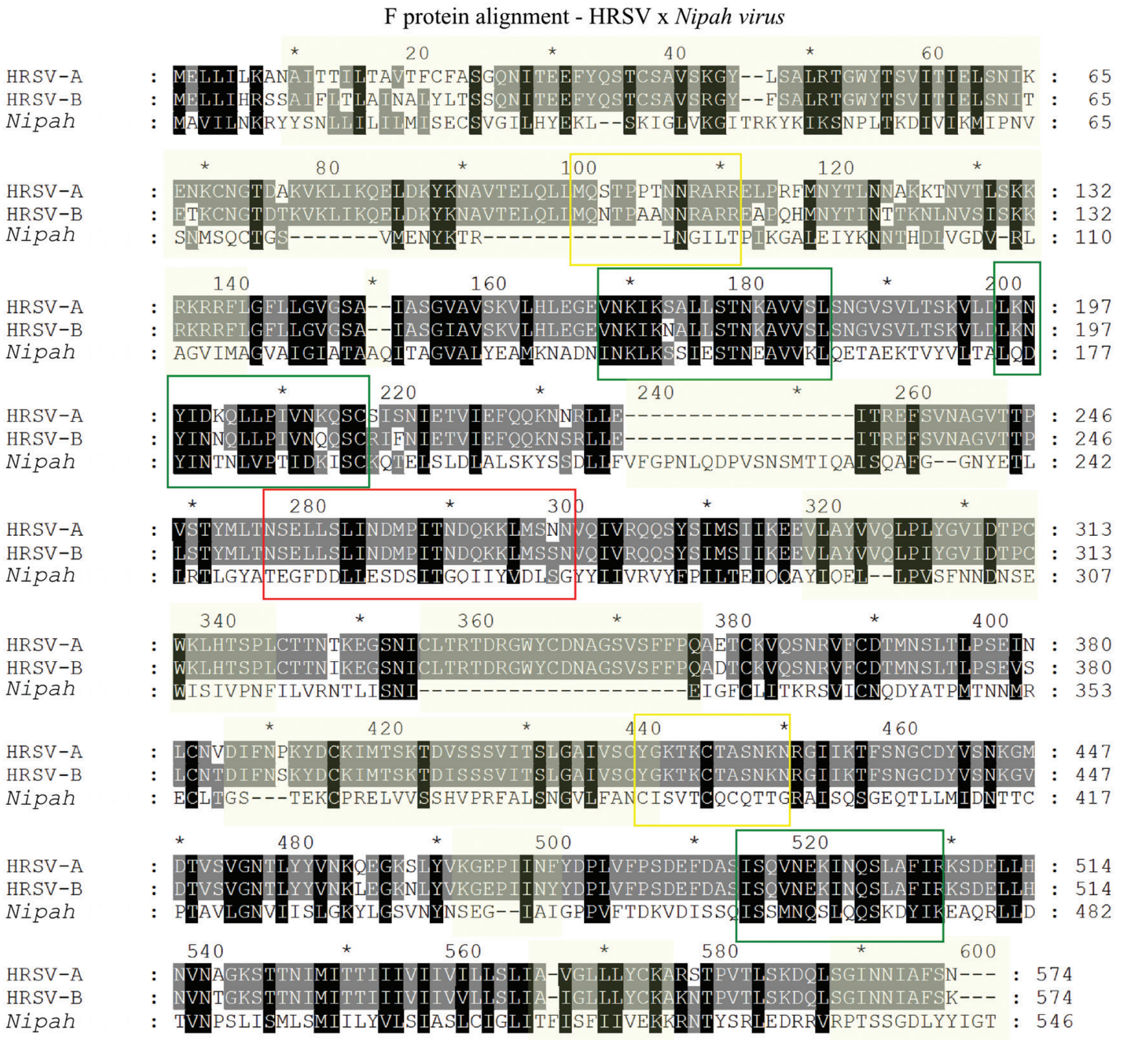


Fig. 6: amino acid (aa) alignment view of the F protein from the selected human respiratory syncytial virus-A (HRSV-A) (P03420.1), HRSV-B (AHJ60043.1) and *Nipah virus* (AAY43915.1) sequences. The green boxes show conserved regions, and the yellow boxes show the possible targets (positions 97-109 and 417-428). The palivizumab-specific antibody epitope sequence is represented by a red box. The ivory, filled boxes represent the unreliable alignment regions according to the PSI-Coffee result and should not be considered.

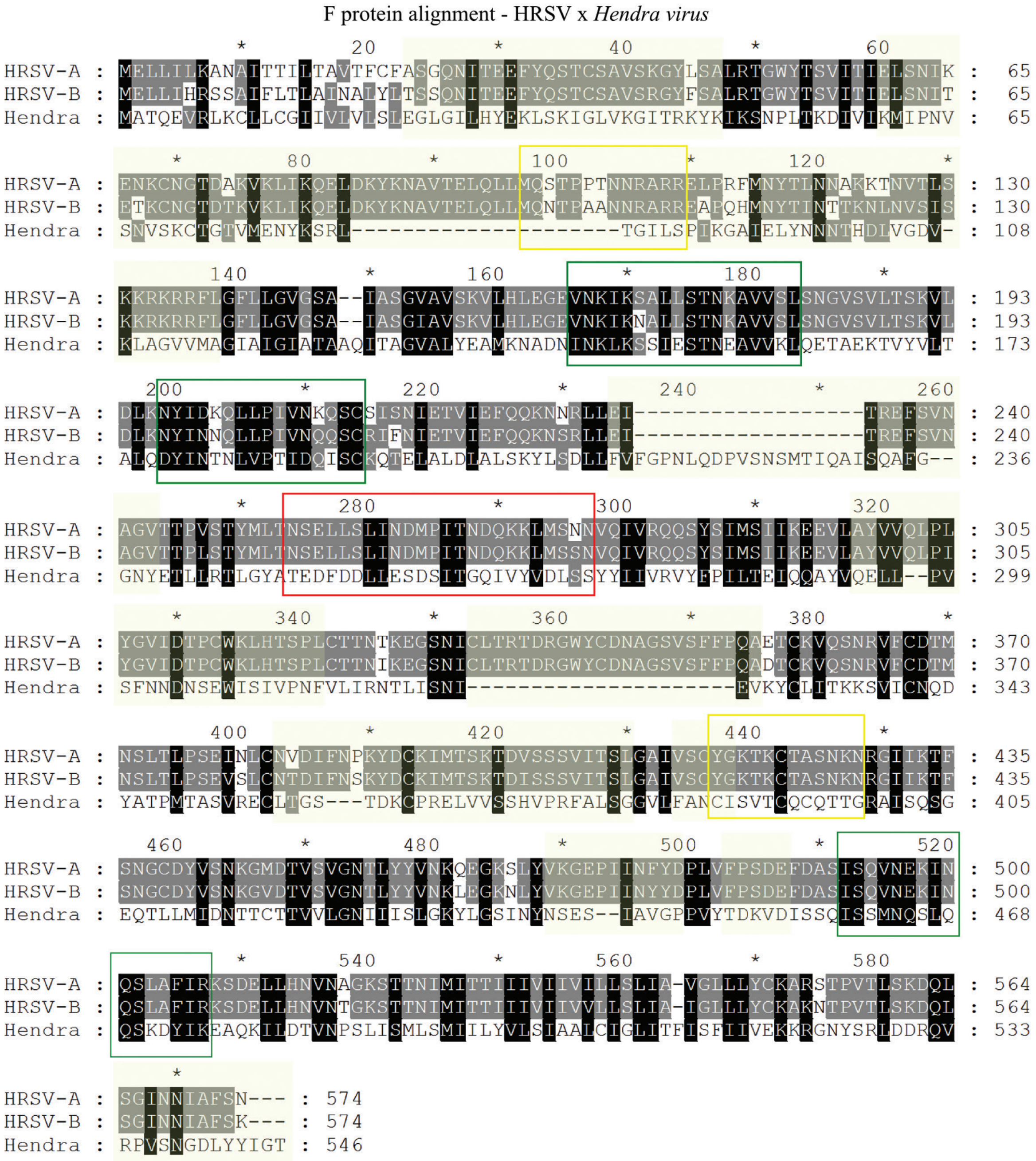


Fig. 7: amino acid (aa) alignment view of the F protein from the selected human respiratory syncytial virus-A (HRSV-A) (P03420.1), HRSV-B (AHJ60043.1) and *Hendra virus* (AEQ38114.1) sequences. The green boxes show conserved regions, and the yellow boxes show the possible targets (positions 97-109 and 417-428). The palivizumab-specific antibody epitope sequence is represented by a red box. The ivory, filled boxes represent the unreliable alignment regions according to the PSI-Coffee result and should not be considered.

F protein alignment- HRSV x *Measles virus*

```
HRSV-A  : MELLILKANAITTILTAVTFCFASGQNITEEFYQST--CSAVSKGYLSALRTGWYTSVITIELSNIK :  65
HRSV-B  : MELLIHRSSAIFLTLAINALYLTSSQNITEEFYQST--CSAVSRGYFSALRTGWYTSVITIELSNIT :  65
Measles : MSIMGLKVNVSAIFMAVLLTLQTPTGQIHWGNLSKIGVVGIGSASYKVMTRSSHQSLV--IKL---- :  61

HRSV-A  : ENKCNGTDAKVKLIKQELDKYKNAVTELQLLMQSTPPTNNRARRELPRFMNYTLNNAKKTNVTLSKK : 132
HRSV-B  : ETKCNGTDTKVKLIKQELDKYKNAVTELQLLMQNTPAANNRARREAPQHMNYTINTTKNLNVSISKK : 132
Measles : ---MPNITLLNNCTRVEIAEYRRLLRTV--------------LEPIRDALNAMTQNIRPVQSVASSR : 111

HRSV-A  : RKRRFLGFLL-----GVG--SAIASGVAVSKVLHLEGEVNKIKSALLSTNKAVVSLSNGVSVLTSKV : 192
HRSV-B  : RKRRFLGFLL-----GVG--SAIASGIAVSKVLHLEGEVNKIKNALLSTNKAVVSLSNGVSVLTSKV : 192
Measles : RHKRFAGVVLAGAALGVATAAQITAGIALHQSMLNSQAIDNLRASLETTNQAIEAIRQAGQEMILAV : 178

HRSV-A  : LDLKNYIDKQLLPIVNKQSCSISNIETVIEFQQKNNRLLEITR----------------EFSVNAGV : 243
HRSV-B  : LDLKNYINNQLLPIVNQQSCRIFNIETVIEFQQKNSRLLEITR----------------EFSVNAGV : 243
Measles : QGVQNYINNELIPSMNQLSCDLIGQKLGLKLLRYYTEILSLFGPSLRDPISAEISIQALSYALGGDI : 245

HRSV-A  : TTPVSTYMLTNSELLSLINDMPITNDQKKLMSNNVQIVRQQSYSIMSIIKEEVLAYVVQLPLYGVID : 310
HRSV-B  : TTPLSTYMLTNSELLSLINDMPITNDQKKLMSSNVQIVRQQSYSIMSIIKEEVLAYVVQLPIYGVID : 310
Measles : NKVLEKLGYSGGDLLGILESRGIKARITHVDTESYFIVLSIAYPTLSEIKG-VIVHRLEGVSYNI-G : 310

HRSV-A  : TPCWKLHTSPLCTTNTKEGSNICLTRTDRGWYCDNAGSVSFFPQAETCKVQSNRVFCDTM------- : 370
HRSV-B  : TPCWKLHTSPLCTTNIKEGSNICLTRTDRGWYCDNAGSVSFFPQADTCKVQSNRVFCDTM------- : 370
Measles : SQEWYTTVPKYVATQGYL--------------------ISNFD-ESSCTFMPEGTVCSQNALYPMSP : 356

HRSV-A  : -NSLTLPSEINLCNVDIFNPKYDCKIMTSKTDVSSSVITSLGAIVSCYGKTKCTASNKNRGIIKTFS : 436
HRSV-B  : -NSLTLPSEVSLCNTDIFNSKYDCKIMTSKTDISSSVITSLGAIVSCYGKTKCTASNKNRGIIKTFS : 436
Measles : LLQECLRGYTKSCARTLVSGSFGNRFILSQGNLIA---NCASILCKCYTTGTIINQDPDKILTYIAA : 420

HRSV-A  : NGCDYVSNKGMDTVSVGNTLYYVNKQEGKSLYVKGEPIINFYDPLVFPSDEFDASISQVNEKINQSL : 503
HRSV-B  : NGCDYVSNKGVDTVSVGNTLYYVNKLEGKNLYVKGEPIINYYDPLVFPSDEFDASISQVNEKINQSL : 503
Measles : DHCPVVEVNGV-TIQVGSRRYPDAVYL---------HRIDLGPPILLERLDVGTNLGNAIAKLEDAK : 477

HRSV-A  : AFIRKSDELLHNVNAGKSTTNIMITTIIIVIIVILLSLIAVGLLLYCKARSTPVTLS--------KD : 562
HRSV-B  : AFIRKSDELLHNVNTGKSTTNIMITTIIIVIIVVLLSLIAIGLLLYCKAKNTPVTLS--------KD : 562
Measles : ELLESSDQILRSMKGLSSTCIVYILIA----VCLGGLIGIPALICCCRGRCNKKGEQVGMSRPGLKP : 540

HRSV-A  : QLSGINNIAFS-N : 574
HRSV-B  : QLSGINNIAFS-K : 574
Measles : DLTGTSKSYVRSL : 553
```

Fig. 8: amino acid (aa) alignment view of the F protein from the selected human respiratory syncytial virus-A (HRSV-A) (P03420.1), HRSV-B (AHJ60043.1) and measles virus (ABY58017.1). The green boxes show conserved regions, and the yellow boxes show the possible targets (positions 97-109 and 417-428). The palivizumab-specific antibody epitope sequence is represented by a red box. The ivory, filled boxes represent the unreliable alignment regions according to the PSI-Coffee result and should not be considered.
